# Cognitive Frailty in Thai Community-Dwelling Elderly: Prevalence and Its Association with Malnutrition

**DOI:** 10.3390/nu13124239

**Published:** 2021-11-25

**Authors:** Mathuramat Seesen, Wachiranun Sirikul, Jetsada Ruangsuriya, Jiranan Griffiths, Penprapa Siviroj

**Affiliations:** 1 Department of Community Medicine, Faculty of Medicine, Chiang Mai University, Chiang Mai 50200, Thailand; mathuramat.s@cmu.ac.th (M.S.); wachiranun.sir@cmu.ac.th (W.S.); 2 Department of Biochemistry, Faculty of Medicine, Chiang Mai University, Chiang Mai 50200, Thailand; jetsada.ruang@cmu.ac.th; 3 Department of Occupational Therapy, Faculty of Associated Medical Sciences, Chiang Mai University, Chiang Mai 50200, Thailand; jiranan.gr@cmu.ac.th

**Keywords:** cognitive frailty, malnutrition, MoCA-B, MNA-SF, serum prealbumin, anthropometry

## Abstract

Cognitive frailty (CF) is defined by the coexistence of physical frailty and mild cognitive impairment. Malnutrition is an underlying factor of age-related conditions including physical frailty. However, the evidence associating malnutrition and cognitive frailty is limited. This cross-sectional study aimed to determine the association between malnutrition and CF in the elderly. A total of 373 participants aged 65–84 years were enrolled after excluding those who were suspected to have dementia and depression. Then, 61 CF and 45 normal participants were randomly selected to measure serum prealbumin level. Cognitive function was assessed using the Montreal Cognitive Assessment-Basic (MoCA-B). Modified Fried’s criteria were used to define physical frailty. Nutritional status was evaluated by the Mini Nutritional Assessment–short form (MNA-SF), serum prealbumin, and anthropometric measurements. The prevalence of CF was 28.72%. Malnourished status by MNA-SF category (aOR = 2.81, 95%CI: 1.18–6.67) and MNA-SF score (aOR = 0.84, 95%CI = 0.74–0.94) were independently associated with CF. However, there was no correlation between CF and malnutrition assessed by serum prealbumin level and anthropometric measurements. Other independent risk factors of CF were advanced age (aOR = 1.06, 95%CI: 1.02–1.11) and educational level below high school (aOR = 6.77, 95%CI: 1.99–23.01). Malnutrition was associated with CF among Thai elderly. High-risk groups who are old and poorly educated should receive early screening and nutritional interventions.

## 1. Introduction

The world is facing the challenge of an increase in the aging population. In 2019, there were 703 million older adults in the world. The number was expected to double to 1.5 billion in 2050. Southeast Asia is one of the regions that had the fastest growth of the aging population [1]. In Thailand, the percentage of the population aged 60 years and above was 17.6% in 2020. Therefore, Thailand has been classified as an aged society. Chiang Mai is a province of northern Thailand with a high proportion of older adults. In 2020, 19.6% of the Chiang Mai population was elderly, which was higher than the national average elderly proportion [2].

Aging is associated with degenerative conditions including sarcopenia [3], cognitive impairment [4], dementia [5], cancer [6], and various chronic non-communicable diseases [7]. Malnutrition is not only a significant factor that worsens age-related conditions but is also a consequence of the aging process [8]. The prevalence of malnutrition was reported to be higher in older compared to younger adults [9]. Older adults are more likely to have determinants of malnutrition, which include loss of appetite [10], loss of taste and smell [11], poor oral health [12], and gastrointestinal problems [13,14,15].

Cognitive frailty is a geriatric syndrome, which has been defined as a combination of mild cognitive impairment (MCI) and frailty without evidence of dementia [16]. MCI is a stage in between normal aging-related cognitive impairments and dementia. Individuals with MCI have a greater degree of cognitive impairment than expected for their age but are otherwise independent [17]. While dementia is defined as a significant cognitive impairment that compromises a person’s daily functioning and is not better explained by another mental disease (e.g., major depressive disorder). To facilitate the detection of cognitive impairment, the instruments designed to screen for cognitive impairment, such as the Mini-Mental State Examination (MMSE) and the Montreal Cognitive Assessment (MoCA), can be useful and have been demonstrated to be effective for different purposes. The MoCA test has been widely acknowledged as a sensitive and effective method for screening MCI, while the MMSE exam is also an accepted tool for screening global cognitive decline or dementia [18]. According to Fried’s criteria [19], frailty is a specific phenotype of a clinical syndrome associated with decreased physiological reserve, increased vulnerability to stressors, and an increased risk of adverse outcomes. In previous research conducted in Asia, the prevalence of cognitive frailty was estimated to be 2.7% in Japan and 1.6% in Singapore [20,21]. In comparison, the prevalence of cognitive frailty was higher in western countries, at 4.4% [22]. Despite its low prevalence in the community, cognitive frailty has been linked to an increased risk of disability, poor health-related quality of life, falling, and death [23,24,25]. Considering cognitive frailty is a reversible state of physical and cognitive impairment, early identification and management of its risk factors is critical for preventing dementia and decline in physical function in the community-dwelling elderly.

However, the underlying mechanisms of cognitive frailty remain uncertain. The strong relationship between physical frailty and cognitive impairment suggests that these conditions share an underlying mechanism, which may include cardiovascular risk factors, chronic inflammation, cerebral vascular disease, neurodegenerative diseases, and malnutrition [26,27,28]. Determining common modifiable factors among cognitive frailty, physical frailty, and cognitive impairment is crucial to developing effective strategies for preventing the progression of disability and dementia among older adults. As mentioned, malnutrition is a well-established, modifiable risk factor of both physical frailty and cognitive impairment. Malnutrition leads to diseases and abnormalities in the elderly namely muscle loss and weakness [29] which cause respiratory [30] and mobility dysfunction [31], impaired immune system [32], increased risk of pressure sores [33], and increased mortality [34]. As reported in the recent study in Chiang Mai Province, Thailand, the prevalence of elderly with nutritional risk was 57.8% [35].

Nutritional status can be evaluated by various methods, including dietary assessment, nutritional assessment tools, clinical evaluations, anthropometric measurement, and laboratory tests. According to an ESPEN consensus, the diagnosis of malnutrition is based on the combination of unintentional weight loss and low body mass index (BMI) or low free fat mass index (FFMI), which is mandatory to fulfill criteria for being defined “at risk” of malnutrition by any validated risk screening tool or measurements [36]. Mini Nutritional Assessment-Short From (MNA-SF) is widely used for evaluating nutritional status in elderly [37,38]. MNA-SF was shown to have a high sensitivity to detect malnutrition and a strong correlation with full Mini Nutritional Assessment in a geriatric population [39,40]. Regarding anthropometric measurement, skinfold thickness was reported to be correlated with fat mass, while calf circumference was associated with muscle mass in older adults [41]. Furthermore, low BMI is widely acknowledged as a diagnostic criterion for malnutrition [36]. Serum prealbumin is one of the laboratory tests that is widely used as an indicator of malnutrition in elderly [42]. It has been proven to be highly correlated with fat free mass in older adults [43]. The use of prealbumin as a nutritional marker of sarcopenia in elderly has been advocated [44].

Although physical frailty, cognitive impairment, and malnutrition are all distinct domains in geriatric syndromes, there is a lot of evidence to support the relationship between malnutrition and physical frailty [45,46,47]. However, studies of the association between malnutrition and cognitive frailty in community-dwelling elderly is limited. Previous studies excluded dementia by self-reported history [21] and the results might not represent the real prevalence since the rate of undetected dementia in older adults was reported to be more than 60% in a community setting [48]. To the best of our knowledge, there is no study of the prevalence of cognitive frailty and its associated factors in community-dwelling elderly in Thailand. Therefore, community-diagnostic research was conducted to characterize the prevalence and possible predictive factors of cognitive frailty in a cross-section study of community-dwelling elderly in Chiang Mai, Thailand.

## 2. Materials and Methods

### 2.1. Study Design and Participants

This cross-sectional study was conducted in July 2021. Participants were community-dwelling older adults aged 65–84 years who lived in Khua Mung Subdistrict, Saraphi District, Chiang Mai Province, Thailand. To identify the eligible participants, the subdistrict primary care unit officers retrieved and examined the aging health data survey of community-dwelling older people aged 65–84 years with independent and partially dependent status in our target area. There were 934 people who were eligible to participate in this study. Additionally, healthy promoting hospital database was reviewed to exclude those who have been diagnosed with dementia, depression, end-stage kidney disease, hepatitis, cirrhosis, autoimmune diseases, cancer, acute trauma, acute illnesses, and those who took steroids. At this stage, 494 older adults were selected by cluster sampling from ten villages. These individuals volunteered to participate in our study after being invited by health care providers and health volunteers who were part of the subdistrict primary health care teams. Twenty-four individuals refused to participate in the study. Four hundred and seventy older adults were screened for dementia and depression and evaluated for cognitive frailty and nutritional status. MSET10 was used to exclude individuals with suspected dementia from the research analysis since cognitive frailty is defined as a combination of mild cognitive impairment (MCI) and frailty without evidence of dementia. Additionally, the TGDS-15 was used to exclude individuals with suspected depression, which can be influenced the assessment of cognitive impairment by the MSET10 and MoCA-B. Fifty-eight and 39 older adults who were suspected to have dementia and depression, respectively, were excluded. Finally, 373 participants were included for the data analysis.

### 2.2. Sample Size Calculation

The total population aged 65–84 years in the study site was 934 persons. The study outcomes include the prevalence of cognitive frailty, the comparison of prealbumin levels between robust and cognitively frail participants and the determination of the associated factors of cognitive frailty. The sample size to study prevalence of cognitive frailty was calculated based on the study of Chye L. et al. [21], which found that the prevalence was 1.6%, a confidence interval of 95%, a bilateral hypothesis test with a significance level of 0.01 and a power of 90%, 494 older adults were enrolled. Due to funding limitations, prealbumin levels were not examined in all eligible participants. For the comparison of prealbumin levels between robust and cognitive frailty, the sample size was calculated based on means and standard deviations of serum prealbumin levels of non-frailty and frailty in the study of Hong, X et al. [49] with a two-sided significance level of 0.05 and a power of 80%. A total of 106 subjects were needed. Considering the rate of participants who might refuse to have a blood test, 15% of subjects were added. A total sample size for the hypothesis testing was 122 participants (61 robust and 61 cognitive frailty individuals).

**Figure 1:**
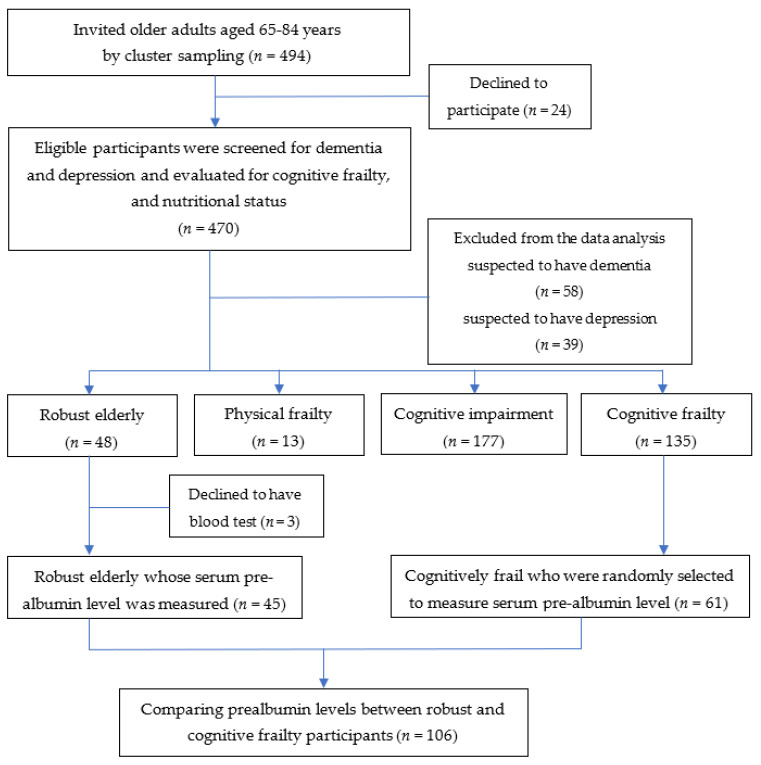
Diagram of participant selection in the study.

After physical and cognitive function examination, there were 135 participants with cognitive frailty, and 48 robust participants. However, only 48 robust individuals were found after screening. Therefore, we recruited all robust individuals to measure prealbumin level. Sixty-one individuals with cognitive frailty were selected by simple random sampling. However, three robust elderlies refused to have a blood test. Therefore, a subset of 61 cognitive frailty and 45 robust participants were tested for serum prealbumin level (see Figure 1).

### 2.3. Questionnaire

The questionnaire consisted of questions on sociodemographic and health information, which included chronic diseases, alcohol consumption, smoking status, physical activity, nutritional status, and activities of daily living (ADL). Participants were interviewed by 10 medical students who were trained and supervised to evaluate cognitive function by an occupational therapist who has The Montreal Cognitive Assessment (MOCA) certification (The certification number is THGRIJI69617-02 and it was given by Dr. Nasreddine, Ziad). Participants were asked whether they had the following diseases: hypertension, type 2 diabetes, chronic kidney disease, coronary artery disease, cerebrovascular disease, dyslipidemia, asthma, COPD, osteoarthritis, and rheumatoid arthritis. The number of diseases was recorded. Frequency of alcohol consumption in the previous 12 months was asked using a chart providing pictures of different types and amount of alcohol beverages. To calculate standard drinks per year, amount of alcohol was converted to numbers of standard drinks, then multiplied by frequency. In addition, the standard drinks per week was calculated to categorize the participants who were “risky alcohol drinking” (greater than ten standard drinks per week) and “non-risky alcohol drinking” (ten standard drinks per week or less). ADL was evaluated using Thai Barthel Index [50], which consisted of ten elements where the maximum score is 20. A higher score represents the more independent functioning of the participants.

### 2.4. Cognitive Frailty Evaluation

Elderly with dementia were excluded using Mental Status Examination Thai 10 (MSET10), which was developed from MMSE Thai 2002 (validated and modified version of the MMSE) [51]. The national study of dementia in Thailand also reported that the using MSET10 for dementia screening outperformed the MMSE-Thai 2002, particularly in poorly educated elderly. Prior to the cognitive function examinations, individuals were interviewed about their reading and writing abilities and education years. Participants with fewer than five years of education were suspected of being illiterate, both in reading and writing, according to the minimum literacy standards set by Thailand’s educational standards in the past. The total score is 29, and the cutoff score for dementia is 22 for individuals who complete elementary school (sensitivity 100.0% and specificity 98.4%), and 17 for those who did not (sensitivity 100.0% and specificity 99.3%). For individuals who are illiterate, the cutoff score is 14 (sensitivity 100.0% and specificity 99.4%). Since depression was indicated to have a negative effect on cognitive testing performance [45], individuals who had depression were excluded using Thai version of the 15-item Geriatric Depression Scale (TGDS-15), which has been proved to be an effective screening tool for major depressive disorder in Thai elderly [52,53]. The maximum score is 15. Participants whose score was ≥6, which is indicative of depression, were excluded from the data analysis.

Cognitive frailty is defined as a presence of both cognitive impairment and physical frailty without dementia [16]. Cognitive impairments were evaluated using Thai version of The Montreal Cognitive Assessment-Basic (MoCA-B), which was an optimized version of the original MoCA test to detect MCI in individuals with illiterate and low education levels. Literacy-dependent tasks were eliminated, and literacy-independent tasks that measured the same cognitive function were substituted. The MoCA-B was validated in community-dwelling Thai elderly people with low education levels and demonstrated excellent discrimination performance for MCI screening (cutoff score of 24, 81% sensitivity and 86% specificity) [54]. The maximum score is 30. The cut-off score for MCI is ≤24. For individuals who had <4 years of education, one point was added to overall score, and two points were added for individuals who had <4 years of education and illiterate.

Physical frailty is defined as a presence of three or more of the following phenotypes, which modified Fried’s criteria [19]. Each component of frailty was assigned a one-point score, and the aggregate scores were used to classify participants as frail (scoring = 3–5), pre-frail (score = 1–2), or robust (score = 0):

(1) Unintentional weight loss: Using this question “In the last year, have you lost more than 4.5 kg unintentionally (i.e., not due to dieting or exercise)?” If yes, then frail for weight loss criterion. At follow-up, weight loss was calculated as: (Weight in previous year—current measured weight)/(weight in previous year) = K. If K ≥ 0.05 and the subject does not report that he/she was trying to lose weight (i.e., unintentional weight loss of at least 5% of previous year’s body weight), then frail for weight loss = Yes. The participants’ weight and height data from the previous year were retrieved from a health promoting hospital records to ensure the accuracy for calculating weight loss.

(2) Weakness: Grip strength was measured using a digital hand dynamometer (TAKEI T.K.K.5401^®^, Takei Scientific Instruments Co., Ltd., Tokyo, Japan). Participants with their shoulder slightly adducted, elbows flexed at 90 degrees, and wrists in neutral posture. The participants were instructed to maintain this posture throughout the test and squeeze the dynamometer with their maximum strength for 2–3 s. The procedure was repeated three times, with a 15-s rest interval between each measurement. The mean value of three trials were reported. Grip strength stratified by gender and body mass index (BMI). Cutoff for grip strength (Kg) criterion for frailty, if she/he met one of the following criteria: BMI ≤ 24 kg/m^2^ and grip strength ≤ 29 kg for men, BMI 24.1–26 kg/m^2^ and grip strength ≤ 30 kg for men, BMI 26.1–28 kg/m^2^ and grip strength ≤ 30 kg for men, BMI > 28 kg/m^2^ and grip strength ≤ 32 kg for men, BMI ≤ 23 kg/m^2^ and grip strength ≤ 17 kg for women, BMI 23.1–26 kg/m^2^ and grip strength ≤ 17.3 kg for women, BMI 26.1–29 kg/m^2^ and grip strength ≤ 18 kg for women, and BMI > 29 kg/m^2^ and grip strength ≤ 21 kg for women.

(3) Slow walking speed: Walk time was measured using the 15-foot walking test. The beginning and finish of the 15-foot (4.57-m) track were marked with adhesive tape, and the time was recorded using a stopwatch. Participants were told to walk at their usual speed. Walk time stratified by gender and height. Cutoff for time to walk 15 feet criterion for frailty, if she/he met one of the following criteria: Height ≤ 173 cm and walk time ≥ 7 s for men, height > 173 cm and walk time ≥6 s for men, height ≤ 159 cm and walk time ≥ 7 s for women, and height >159 cm and walk time ≥6 s for women.

(4) Self-reported exhaustion: Using the following, two statements are read. (a) I felt that everything I did was an effort; (b) I could not get going. The question is asked “How often in the last week did you feel this way?” 0 = rarely or none of the time (<1 day), 1 = some or a little of the time (1–2 days), 2 = a moderate amount of the time (3–4 days), or 3 = most of the time. Subjects answering “2” or “3” to either of these questions are categorized as frail.

(5) Low physical activity level: The physical activity was measured using Thai version of the short format International Physical Activity Questionnaire (IPAQ), which was reported to have acceptable validity and reliability when compared to other physical activity tools used in epidemiological studies [55]. Kcal per week was calculated. Men and women who had physical activity <383 and <270 Kcal per week, respectively, are categorized as frail.

In our study, cognitive frailty is defined as a presence of both MCI (defined as MoCA-B score of 24 or below) and physical frailty by a presence of three or more points in Fried’s criteria. Participants who had MCI without physical frailty are categorized into the MCI group, whereas those who had physical frailty without MCI are classified as physical frailty. Robust participants are characterized by the absence of both MCI and frailty.

### 2.5. Nutritional Status Evaluation

Four measurement methods were used to evaluate nutritional status in our study as follows:

#### 2.5.1. Mini Nutritional Assessment Short-Form (MNA-SF)

Participants were interviewed by the examiners [39,40]. The maximum score is 14. The score is interpreted as follow: 12–14 indicates “normal nutritional status”, 8–11 indicates “at risk of malnutrition”, and <8 indicates “malnourished”.

#### 2.5.2. Serum Prealbumin Level

Prealbumin levels were analyzed using the ELISA technique. Upon consent, 5 mL blood was drawn into a heparinized blood collection tube from robust and cognitive frail subjects. Plasma was centrifuged at 1000× *g*, 25 °C for 15 min. The plasma in supernatant was carefully aspirated into a 1.5 mL Eppendorf tube and stored in −80 °C for prealbumin analysis.

Prealbumin concentration was determined by a commercial ELISA kit (Boster Biological Technology, Pleasanton, CA, USA; EK1684) following the manufacturer’s protocol. Briefly, the plasma samples were completely thawed at 25 °C and diluted with deionized water along with the kit assay diluent to fit the kit standard curve. Then, 100 μL of diluted samples and standards were loaded into each ELISA well plate in duplicate and the plate was incubated at 37 °C for 90 min. Then, the solution in each well was replaced with biotinylated prealbumin antibody, washed, replaced with avidin-biotin-peroxidase complex, washed again, replaced with TMB substrate, and topped up with stop solution. The absorbance at 450 nm was determined and the concentrations of the prealbumin in the plasma samples were calculated from the corresponding standard curve. The prealbumin level <200 mg/L was categorized as low prealbumin [56].

#### 2.5.3. Triceps Skinfold Thickness

Triceps skinfold thickness was measured using Hapenden skinfold caliper to the nearest 0.1 cm by a single investigator. The participants were measured at the posterior midpoint of right upper arm between the acromion process and the olecranon process while standing [57].

#### 2.5.4. Calf Circumference

The maximum calf circumference was measured to the nearest 0.1 cm using a measuring tape while participants were standing, without compression of the subcutaneous tissue. The measurement was performed by a single investigator. A circumference of <34 cm in men and <33 cm in women was categorized as a low calf circumference [58].

#### 2.5.5. Body Mass Index (BMI)

BMI was calculated as weight in kilograms divided by the square of the height in meters (kg/m^2^). According to the Asia-Pacific regional guideline on BMI for Asian people, the cut-off point of <18.5 kg/m^2^ indicated underweight [59].

### 2.6. Statistical Analysis

Sociodemographic data and the prevalence of malnutrition in different physical frailty and cognition categories were analyzed using chi-square test for categorical variables. For continuous variables, one-way ANOVA for parametric distributed data and the Kruskal–Wallis test for non-parametric distributed data were performed with a post-hoc pairwise comparison test. The comparison of mean prealbumin levels between robust and cognitively frail participants was performed by the Rank-sum test. Multiple logistic regression analysis was performed to explore the association between nutritional status, which was measured by four methods, and cognitive frailty with potential confounders adjustment, including age, gender, marital status, living status, educational levels, number of underlying diseases, alcohol consumption, smoking status, and ADL score. Furthermore, a full exploratory model of a significant nutritional status parameter with pre-specified confounders by multivariable logistic regression was presented to determine the associated factors of cognitive frailty. A *p*-value < 0.05 was considered statistically significant. The results of this study were reported according to the Strengthening of the Reporting of Observational Studies in Epidemiology (STROBE) checklist.

### 2.7. Ethical Considerations

All subjects gave their informed consent for inclusion before they participated in the study. The study was conducted in accordance with the Declaration of Helsinki, and the protocol was approved by the Ethics Committee of Faculty of Medicine, Chiang Mai University (Ethical number: COM-2564- 08031: Date of approval; 22 April 2021).

## 3. Results

### 3.1. Socio-Demographic Information of Robust, Physical Frailty MCI, and Cognitive Frailty in the Elderly

The characteristics of participants by physical frailty and cognitive status are presented in Table 1. One hundred and thirty five cognitively frail participants from 470 participants were examined. The prevalence was 28.72%. The mean age of participants was 70.45 years. A majority of the subjects were female (58.4), married (63.8), living with others (88.7%), had an education level of grade 4–6 (50.4%), and non-smokers (93.3%). The majority of them had one to two underlying diseases (61.1%) or no underlying disease (28.7%). Only 38 patients (10.2%) had three or more underlying diseases. Regarding ADL, all older adults were independent as their scores were higher than 11. The mean ADL score was 19.63 ± 0.83. Moreover, there was no significant difference in the proportions of specific underlying diseases in different physical frailty and cognition statuses. The proportion of risky alcohol consumption (greater than 10 standard drinks per week) also significantly varied among robust (18.75%), MCI (11.9%), physical frail (7.7%) and cognitively frail (5.2%) participants (*p* = 0.011). Overall, the elderly who had cognitive frailty were older (*p* < 0.001) and had lower education (*p* < 0.001). In addition, elderly with MCI were older than robust participants (*p* < 0.001). Compared to robust participants, elderly who had MCI and cognitive frailty consumed fewer standard drinks of alcohol (*p* < 0.01).

### 3.2. Nutritional Status of Robust, Physical Frailty MCI, and Cognitive Frailty in the Elderly

The prevalence of malnutrition in elderly people with different physical and cognitive statuses is presented in Table 2. The prevalence of malnourished measured by MNA-SF was highest in the physical frailty group (*p* = 0.04). However, the prevalence of underweight elderly was not different between the four groups. There was no difference of prealbumin levels measured in robust and cognitively frail participants as shown in Table 3. Moreover, triceps skinfold thickness and calf circumference were similar in both groups.

### 3.3. Correlation Coefficient among Nutritional Status Measured by Different Methods

The correlation coefficients among nutritional status measured by different methods are presented in Table 4. The score of MNA-SF was positively correlated with triceps skinfold thickness (r = 0.262, *p* < 0.001), and calf circumference (r = 0.304, *p* < 0.001). In addition, triceps skinfold thickness was positively correlated with calf circumference (r = 0.212, *p* < 0.001). On the contrary, serum prealbumin levels were not correlated with triceps skinfold thickness or calf circumference.

### 3.4. Association of Malnutrition and Cognitive Frailty

Using the unadjusted analysis, increased calf circumference and MNA-SF scores decreased the risk of cognitive frailty, whereas a malnourished status determined by the MNA-SF category increased the risk of cognitive frailty. The association between malnourished status by MNA-SF, MNA-SF scores and cognitive frailty remained significant after adjusting for potential confounders, including age, gender, marital status, living status, educational levels, number of chronic diseases, alcohol consumption, smoking status, and ADL score. However, prealbumin levels, triceps skinfold thickness, and calf circumference were not associated with cognitive frailty. The results from univariable (model 1) and multivariable logistic regression (model 2) are provided in Table 5.

### 3.5. Factors Associated with Cognitive Frailty

Factors associated with cognitive frailty are shown in Table 6. Increased age (aOR = 1.06, 95%CI: 1.02–1.11), educational level below high school (aOR = 6.77, 95%CI: 1.99–23.01), and malnourished status by MNA-SF (aOR = 2.81, 95%CI: 1.18–6.67) significantly increased the risk of cognitive frailty after adjusting for gender, marital status, living status, number of chronic diseases, alcohol consumption, smoking status, and ADL score. The number of alcohol standard drinks per year and ADL score also showed a borderline significant association with cognitive frailty.

## 4. Discussion

To the best of our knowledge, this is the first study to determine the prevalence of cognitive frailty and the association with nutritional status in Thai community-dwelling elderly. Overall, the prevalence of cognitive frailty in our study is 28.72%. Furthermore, the association of malnutrition which was evaluated by MNA-SF and cognitive frailty was demonstrated. In this study, cognitive impairment was defined using MoCA-B. The following provides an explanation for the MoCA-B used to screen for MCI in our study. The MoCA-B has an advantage over other mild cognitive impairment assessments in that it was modified and validated for the purpose of screening for MCI in elderly Thai people who are illiterate and poorly educated (educational years less than 5 years), since the majority of the community-dwelling elderly Thai population have low education levels [36]. Additionally, as mentioned previously in the method, the MoCA-B demonstrated excellent discrimination performance for MCI screening in this population. In comparison to other validated assessments (e.g., the MMSE or the Mini-Cog test), the majority of them are aimed at detecting cognitive impairments or dementia and need literacy skills. On the other hand, MSET10, which was a modified and validated version of MMSE-Thai 2002, was used for different purposes due to its specificity for detecting dementia. Moreover, the advantage of using MSET10 compared with MMSE-Thai 2002 is that it provides better specificity and negative predictive value for detecting suspected dementia in poorly educated elderly people.

The prevalence of cognitive frailty in our study is higher than the previous study in Singapore [21] in which the prevalence was 1.6%. This could be explained by the younger participants in the Singapore study, who were adults aged 55 years and above, since the prevalence of cognitive frailty increases with age [25]. Besides population age, the difference in prevalence could be explained by educational level, which was lower in our study. A higher level of education was reported to be correlated with higher cognitive function and slower cognitive decline in the elderly [60,61]. Compared to a study in Hong Kong [62], our prevalence is lower since that study included participants from residential services who were functionally independent, and those who had depression. Moreover, the criteria used to define cognitive frailty in this study were different from our study as the authors included pre-physical frailty.

Cognitive frailty participants had a lower educational level. This group had the smallest percentage of who had been educated beyond primary school. This result is consistent with the previous reports, which indicated that more years of education in community-dwelling older adults correlated with higher cognitive levels and slower cognitive decline [60,61]. Besides cognitive function, limited education was reported to be a risk factor of frailty [60,63]. The relationship between lower education and frailty status could be explained by the lower opportunity to work which is a protective factor against decrease in ADL [64]. Moreover, higher education leads to the higher health literacy, which was indicated to be associated with non-frailty [65].

Regarding alcohol consumption, elderly with MCI were reported to have significantly higher levels compared to the robust group. The reason behind this result could be explained by the neurotoxic effect of alcohol [66]. This result is similar to the previous study, which reported the correlation of alcohol consumption and higher risk of MCI [47,67]. Interestingly, alcohol consumption in elderly with cognitive frailty was significantly lower than robust participants. This result is consistent with several longitudinal studies, which demonstrated an inverse correlation between alcohol intake and frailty risk [68,69]. This might be due to several factors including “sick quitter” effect, survival bias, residual confounding, and the reverse causality which is a limitation of cross-sectional study. Notably, asking participants with mild cognitive impairment and cognitive frailty to report their alcohol consumption within the previous year may be unreliable as their ability to recall accurately may be impaired.

Although dietary assessment and clinical evaluation are used to evaluate nutritional status, we could not apply these in our study due to their limitations. Regarding dietary assessment, it might not be suitable for our population since the results depend on participants’ memory [70]. We did not perform clinical evaluation since it requires clinical experience of the examiners. Therefore, MNA-SF, anthropometric, and laboratory measurements were used in our study. For anthropometric measurement, we used triceps skinfold thickness and calf circumference since they are indirect methods to assess fat and muscle mass in elderly [36]. Moreover, low BMI is accepted as a diagnostic criterion for malnutrition [36]. Our study used serum prealbumin level as a biomarker for malnutrition because of its correlation with fat free mass [43], which have been shown to be associated with frailty in older adults [71].

The prevalence of elderly with abnormal MNA-SF in our study is similar to the previous study in Thailand which reported that 57.8% of community-dwelling older adults were at risk of malnutrition and malnourished [35]. Compared to robust and MCI groups, physical frailty and cognitive frailty groups showed lower MNA-SF score, which is consistent with the previous study [21]. This could be explained by the influence of malnutrition which has greater effects on physical strength than cognition [72]. This reason might also account for the greater percentage of malnourished participants in the physical frailty group. Additionally, nutritional status was shown to have a significant mediating effect on the relationship between sarcopenia, which has high rate of coexistence with physical frailty and share similar criteria with physical frailty [73,74], and cognitive function [75]. Moreover, our study indicated the association of malnutrition, as measured by MNA-SF and cognitive frailty in older adults which is consistent with other previous studies [21,62].

Prealbumin levels were reported to be correlated with MNA-SF in geriatric population [39]. However, we could not demonstrate a difference in serum prealbumin levels between robust and cognitive frail groups, nor an association of prealbumin levels and cognitive frailty. The percentages of those who had low albumin level in our study were higher than previous studies in Singapore and Belgium [76,77], even in robust elderly. This could be explained by the difference in living standards in our setting. This lack of association might be caused by the number of robust participants, whose measured prealbumin levels were lower than expected.

Our study was unable to demonstrate the correlation between cognitive frailty and nutritional status evaluated by anthropometric measurement which includes triceps skinfold thickness, calf circumference, and BMI. These results are in agreement with previous study of nutrition al status in the geriatric population [78]. The reason behind these findings could be the limitation of these methods because of the changes in body composition during ageing [79]. In older adults, skinfold thickness measurements were indicated to be less accurate due to age-related increases in adiposity, changes in skin elasticity, hydration, subcutaneous adipose tissue compressibility, and muscle tone [80]. BMI threshold also has limitations to detect malnutrition in the elderly since it was reported to overestimate the number of overweight individuals [81]. Additionally, the association between calf circumference, which represents muscle mass in the elderly [82,83], and cognitive frailty could not be observed after adjusting the confounders.

Apart from nutritional status, factors associated with cognitive frailty in our study are old age and low educational level. Our results demonstrated that age is a risk factor of cognitive frailty which is consistent with previous studies [25,84]. Regarding educational level, for the abovementioned reasons, education is known to affect cognition and frailty through work and health literacy.

In nutritional status measurements, we observed that the MNA-SF score was positively correlated with triceps skinfold thickness and calf circumference. This finding is similar to the previous study in a community setting in southern Thailand [85] and Ethiopia [86]. However, serum prealbumin levels were not correlated with other measurement methods. These results contradict previous studies in hospitalized older adults [43,87], which could be explained by the different physical statuses of the participants. In addition, another reason for the lack of an association might be the inadequate number of subjects whose prealbumin levels were measured in our study.

The strength of this study is the use of cluster sampling of the participants. Moreover, we excluded participants who were highly suspected to have dementia and depression which causes an incorrect diagnosis of cognitive frailty. However, there are several limitations. Firstly, as a cross-sectional study, a causal relationship cannot be indicated. Secondly, the sample size was too small to demonstrate a significant difference between nutritional status measured by serum prealbumin levels and cognitive frailty. In addition, the numbers of question were limited to prevent a decline in the participants concentration. Therefore, several factors that are related cognitive frailty and malnutrition which includes medications, household income, appetite, and gastrointestinal problems, were not evaluated. The absence of medication information in the multivariable analysis is undoubtedly another limitation of our study. Multiple mechanisms support the association between polypharmacy and malnutrition. Long-term usage of several medications results in anorexia, which is typically a mild to severe impairment of the gastrointestinal system. Additionally, many medications have the potential to adversely affect nutritional status by affecting taste perception, intestinal absorption, and metabolism, or by causing the loss of essential vitamins and minerals [88]. According to a survey of spontaneous adverse drug reactions (ADRs), taste alteration was found in 75% of instances [89]. The leading causes were angiotensin-converting enzyme inhibitors (ACEI) and HMG-CoA reductase inhibitors (statins), which are commonly prescribed for hypertension and dyslipidemia patients. In addition, as reported in a recent systematic review [90], adverse drug reactions, including drug–drug interactions and drug–nutrient interactions, can influence nutritional status, frailty, and cognitive impairment when using between two and 11 medications. For example, frailty and cognitive impairment can be caused by the drug–nutrient interactions of statins on coenzyme Q_10_ deficiency and metformin on vitamin B_12_ deficiency. Future research on the relationship between malnutrition and cognitive frailty should consider the confounding effects of polypharmacy and adverse drug reactions. Nonetheless, we presumed that the polypharmacy and medicine used in our study population might not be substantially different between the comparison groups and had a modest effect on cognitive frailty and nutritional status. Since there was no significant difference in the number of underlying disorders or the proportion of specific underlying diseases.

## 5. Conclusions

We demonstrated the association between malnutrition measured by MNA-SF and cognitive frailty among the community-dwelling elderly. Advanced age and low educational level were observed to be risk factors of cognitive frailty. Early screening and nutritional interventions to prevent the worsening of cognitive frailty should be implemented in these groups. In addition, further studies to examine biomarkers or anthropometric measurements to detect cognitive frailty early in community-dwelling older adults with an adequate sample size should be conducted.

## Figures and Tables

**Table 1:** Socio-demographic characteristics of participants according to physical frailty and cognitive status.

Characteristics (Mean ± SD)/*n* (%))	Total (*n* = 373)	Robust ^a^ (*n* = 48)	Physical Frailty ^b^ (*n* = 13)	MCI ^c^ (*n* = 177)	Cognitive Frailty ^d^ (*n* = 135)	*p*-Value
Age (years), mean ± SD	70.45 ± 5.40	68.17 ± 3.06	69.08 ± 3.64	70.15 ± 4.84	71.78 ± 6.47	<0.001 *^,ac,ad,bd,cd^
Gender Male Female	155 (41.6) 218 (58.4)	26 (54.2) 22 (45.8)	3 (23.1) 10 (76.9)	73 (41.2) 104 (58.8)	53 (39.3) 82 (60.7)	0.15
Marital status Married Single/divorced/ Widowed	238 (63.8) 135 (36.2)	37 (77.1) 11 (22.9)	9 (69.2) 4 (30.8)	108 61.0) 69 (39.0)	84 (62.2) 51 (37.8)	0.20
Living alone	42 (11.3)	5 (10.4)	2 (15.4)	23 (13.0)	12 (8.9)	0.67
Educational level No education Grade 1–3 Grade 4–6 High school/Vocational certificate Bachelor’s degree	8 (2.1) 138 (37.0) 188 (50.4) 24 (6.4) 15 (4.0)	1 (2.1) 9 (18.8) 18 (37.5) 12 (25.0) 8 (16.7)	1 (7.7) 6 (46.2) 4 (30.8) 1 (7.7) 1 (7.7)	4 (2.3) 60 (33.9) 99 (55.9) 9 (5.1) 5 (2.8)	2 (1.5) 63 (46.7) 67 (49.6) 2 (1.5) 1 (0.7)	<0.001 **
Numbers of underlying diseases						
0 1–2 ≥3	107 (28.7) 228 (61.1) 28 (10.2)	14 (29.2) 28 (58.3) 6 (12.5)	4 (30.8) 9 (69.2) -	57 (32.2) 105 (59.3) 15 (8.5)	32 (23.7) 86 (63.7) 17 (12.6)	0.512
Underlying diseases, n (%)						
No underlying disease Hypertension Type 2 Diabetes mellitus Dyslipidemia Gout Thyroid diseases Coronary heart disease Stroke Chronic kidney disease Osteoarthritis	103 (27.6) 195 (52.3) 58 (15.6) 59 (15.8) 16 (4.3) 12 (3.2) 12 (3.2) 11 (3.0) 10 (2.7) 8 (2.1)	14 (29.2) 20 (41.7) 7 (14.6) 10 (20.8) 3 (6.3) 1 (2.1) 1 (2.1) 2 (4.2) - 1 (2.1)	4 (30.8) 7 (53.9) 2 (15.4) - - - - - - 1 (7.7)	55 (31.1) 92 (52.0) 21 (11.9) 29 (16.4) 7 (4.0) 7 (4.0) 4 (2.3) 6 (3.4) 5 (2.8) 2 (1.1)	30 (22.2) 76 (56.3) 28 (20.7) 20 (14.8) 6 (4.4) 4 (3.0) 7 (5.2) 3 (2.2) 5 (3.7) 4 (3.0)	0.37 0.383 0.201 0.322 0.780 0.809 0.418 0.798 0.525 0.361
Alcohol drinking in previous year						
Risky alcohol drinking (>10 standard drinks per week) Non-risky alcohol drinking (≤10 standard drinks per week) No alcohol drinking	38 (10.2) 20 (5.4) 315 (85.4)	9 (18.75) 3 (6.25) 36 (75.0)	1 (7.7) 2 (15.4) 10 (76.9)	21 (11.9) 12 (6.8) 144 (81.3)	7 (5.2) 3 (2.2) 125 (92.6)	0.011 *
Current smoking	25 (6.7)	3 (6.3)	0 (0)	14 (7.9)	8 (5.9)	0.68
ADL score, mean ± SD	19.63 ± 0.83	19.83 ± 0.48	19.58 ± 0.79	19.66 ± 0.80	19.53 ± 0.94	0.17

MCI, Mild cognitive impairment; ADL, Activities of daily living; SD, Standard deviation; All *p*-values of the categorical Variables were obtained from Chi-square test, for the continuous variables, *p*-values were obtained from one-way ANOVA, for the non-parametric continuous variables, *p*-values were obtained Kruskal-Wallis test; Statistically significant for *p* ≤ 0.05, ** Statistically significant for *p* ≤ 0.001; The comparison groups were denoted as following ^a^ Robust, ^b^ Physical Frailty, ^c^ MCI, and ^d^ Cognitive Frailty; Statistically significant (*p* ≤ 0.05) for post-hoc pairwise comparison was denoted as following, ^ab^ Robust and physical frailty, ^ac^ Robust and MCI, ^bd^ Physical frailty and cognitive frailty, ^cd^ MCI and cognitive frailty.

**Table 2:** Nutritional status of robust, physical frailty MCI, and cognitive frailty in the elderly.

Nutritional Status	Total (n = 373)	Robust ^a^ (n = 48)	Physical Frailty ^b^ (n = 13)	MCI ^c^ (n = 177)	Cognitive Frailty ^d^ (n = 135)	*p*-Value
MNA-SF, n (%) At risk of malnutrition Malnourished	221 (59.2) 31 (8.3)	30 (62.5) 2 (4.2)	6 (46.2) 3 (23.1)	106 (59.9) 8 (4.5)	79 (58.5) 18 (13.3)	0.04 *
MNA-SF score, mean ± SD	10.41 ± 1.84	10.79 ± 1.51	9.69 + 2.18	10.68 + 1.62	10.02 + 2.10	<0.01 *^,ab,ad,bc,cd^
Triceps skinfold thickness (cm), mean ± SD	18.17 ± 8.79	19.41 ± 8.14	22.86 ± 15.97	17.25 ± 7.17	18.49 + 9.84	0.08
Low calf circumference, n (%)	194 (52)	17 (35.4)	6 (46.2)	96 (54.2)	75 (55.6)	0.09
Calf circumference (cm), mean ± SD	33.09 ± 4.55	34.20 ± 2.95	33.47 ± 3.55	33.32 ± 5.53	32.36 ± 3.48	0.08
Underweight by BMI, n (%)	42 (11.3)	3 (6.3)	1 (7.7)	20 (11.3)	18 (13.3)	0.58

MCI, Mild cognitive impairment; MNA-SA, Mini Nutritional Assessment Short-Form; SD, Standard deviation; All *p*-values of the categorical variables were obtained from Chi-square test, for the parametric continuous variables, *p*-values were obtained from one-way ANOVA, for the non-parametric continuous variables, *p*-values were obtained Kruskal-Wallis test; * Statistically significant for *p* ≤ 0.05; The comparison groups were denoted as following ^a^ Robust, ^b^ Physical Frailty, ^c^ MCI, and ^d^ Cognitive Frailty; Statistically significant (*p* ≤ 0.05) for post-hoc pairwise comparison was denoted as following, ^ab^ Robust and Physical frailty, ^ac^ Robust and MCI, ^bd^ Physical frailty and Cognitive frailty, ^cd^ MCI and Cognitive frailty.

**Table 3:** Comparison of prealbumin levels between robust and cognitive frailty in the elderly.

Nutritional Status	Total (n = 106)	Robust (n = 45)	Cognitive Frailty (n = 61)	*p*-Value
Low-prealbumin level, n (%)	95 (89.6)	39 (86.7)	56 (91.8)	0.162
Prealbumin levels (mg/L), median (IQR)	85.69 (92.90)	85.8 (89.70)	85.62 (92.84)	0.501

*p*-values of the categorical variables were obtained from Chi-square test, for the continuous variables, *p*-value were obtained from Rank-sum test.

**Table 4:** Pearson’s correlation coefficient among nutritional status measured by different methods including MNA-SF, serum prealbumin, triceps skinfold thickness, and calf circumference.

Pearson’s Correlation Coefficient	Nutritional Status Evaluation		
	Prealbumin Levels	Triceps Skinfold Thickness	Calf Circumference
MNA-SF	−0.005	0.262 **	0.304 **
Prealbumin levels	-	0.036	0.045
Triceps skinfold thickness	-	-	0.212 **

MNA-SA, Mini Nutritional Assessment Short-Form; ** Statistically significant for *p* ≤ 0.001.

**Table 5:** Association of malnutrition and cognitive frailty divided by different measurement methods.

Measurement Methods	Model 1		*p*-Value	Model 2		*p*-Value
	Crude OR	95% CI		Adjusted OR	95% CI	
MNA-SF category						
At risk of malnutrition	1.23	0.76–2.00	0.391	1.28	0.77–2.11	0.343
Malnourished	3.24	1.41–7.42	<0.01 **	2.81	1.18–6.67	0.019 *
MNA-SF score	0.84	0.74–0.94	<0.01 **	0.84	0.75–0.96	<0.01 **
Prealbumin levels	1.00	0.99–1.00	0.240	1.00	0.99–1.00	0.222
Triceps skinfold thickness	1.01	0.98–1.03	0.600	1.01	0.98–1.04	0.268
Calf circumference	0.92	0.86–0.98 *	0.010 *	0.93	0.86–1.01	0.090
Underweight by BMI	1.37	0.71–2.63	0.340	1.47	0.69–3.13	0.320

Model 1: unadjusted; Model 2: adjusted for age, gender, marital status, living status, educational levels, number of underlying diseases, alcohol consumption, smoking status, and ADL score; MCI, Mild cognitive impairment; MNA-SA, Mini Nutritional Assessment Short-Form; ADL, Activities of daily living; BMI, Body mass index; Crude OR, Crude odds ratio from univariable logistic regression; Adjusted OR, Adjusted odds ratio from multivariable logistic regression; CI, Confidence interval; Underweight by BMI, BMI ≤ 18.5 kg/m^2^; * Statistically significant for *p* ≤ 0.05; ** Statistically significant for *p* ≤ 0.01.

**Table 6:** Full exploratory model of factors associated cognitive frailty in community-dwelling elderly.

Variables	Adjusted OR	95% CI	*p*-Value
Age	1.06	1.02–1.11	<0.01 **
Female	0.88	0.52–1.47	0.358
Educational level above high school	6.77	1.99–23.01	<0.01 **
Married	1.12	0.65–1.95	0.680
Living alone	0.60	0.26–1.37	0.228
Number of underlying diseases	1.17	0.94–1.46	0.172
Alcohol drinking in previous year (Total of standard drinks per week)	1.00	0.99–1.00	0.051
Smoking status	0.83	0.31–2.24	0.712
ADL score	0.78	0.59–1.02	0.070
MNA-SF			
At risk of malnutrition	1.28	0.77–2.11	0.343
Malnourished	2.81	1.18–6.67	0.019 *

ADL, Activities of daily living; MNA-SA, Mini Nutritional Assessment Short-Form; Adjusted OR, Adjusted odds ratio from multivariable logistic regression; OR, Odds ratio; CI, Confidence interval; * Statistically significant for *p* ≤ 0.05; ** Statistically significant for *p* < 0.01.

## Data Availability

The data presented in this study are available on request from the correspondent author.
